# Biotemplated Artificial Olive Leaf-Structured TiO_2_ Decorated with Pt and Au for Enhanced Photocatalytic Hydrogen Production

**DOI:** 10.3390/biomimetics11050300

**Published:** 2026-04-26

**Authors:** Juan Martín-Gómez, Jesús Hidalgo-Carrillo, M. Carmen Herrera-Beurnio, Alejandro Ariza-Pérez, Alberto Marinas, Francisco J. Urbano

**Affiliations:** Departamento de Química Orgánica, Instituto Químico para la Energía y el Medioambiente (IQUEMA), Universidad de Córdoba, E-14071 Córdoba, Spain; q92magoj@uco.es (J.M.-G.); jesus.hidalgo@uco.es (J.H.-C.); b52hebem@uco.es (M.C.H.-B.); q82arpea@uco.es (A.A.-P.); alberto.marinas@uco.es (A.M.)

**Keywords:** biotemplated TiO_2_, olive leaf template, noble metal photodeposition, glycerol photoreforming, hydrogen production

## Abstract

Biotemplated strategies inspired by natural architecture have emerged as an effective strategy to improve the performance of photocatalytic materials. In this work, TiO_2_-based photocatalysts were synthesized using olive leaves as a biological template to reproduce their hierarchical microstructure and enhance photocatalytic hydrogen production. The artificial olive leaf (AOL) support was obtained through a biotemplated ion-exchange process followed by hydrolysis and calcination. It was then modified by photodeposition of Au or Pt nanoparticles. The materials were characterized by SEM, XRD, N_2_ adsorption–desorption, UV–Vis spectroscopy, and XPS to evaluate their structural and optical properties. SEM confirmed the successful replication of both the external morphology and internal architecture of the olive leaf, while XRD revealed low crystallinity with anatase as the only TiO_2_ phase. Optical characterization showed a reduced band gap (~2.97 eV), and extended absorption toward the visible region, with Au nanoparticles exhibiting a plasmonic band at ~550 nm, whereas Pt enhanced light-harvesting efficiency. XPS indicated the presence of oxygen vacancies and Ti^3+^ species that promote metal–support interactions. Photocatalytic glycerol photoreforming showed a strong enhancement in hydrogen production after noble metal incorporation, reaching up to 14-fold under UV irradiation and 23-fold under simulated solar light for the Pt-modified catalyst, highlighting the synergy between biotemplated structuring and noble metal deposition.

## 1. Introduction

The growing global demand for sustainable and carbon-neutral energy sources has intensified research into alternative fuels capable of mitigating greenhouse gas emissions and reducing dependence on fossil fuels. In this context, hydrogen is considered one of the most promising energy carriers because of its high energy content (120 MJ kg^−1^), which is significantly higher than that of conventional fuels such as gasoline (44.4 MJ kg^−1^). In addition, its oxidation produces only water, with no direct CO_2_ or other atmospheric pollutant emissions [1,2].

Among the various routes for sustainable hydrogen production, semiconductor-based photocatalytic processes have attracted considerable attention, as they enable the direct conversion of solar energy into chemical energy under mild operating conditions. Although photocatalytic water splitting has been extensively studied [3,4], this process has thermodynamic and kinetic limitations that significantly reduce its practical efficiency. As an alternative, the photocatalytic reforming of oxygenated organic compounds has emerged as a particularly promising strategy. This process involves treating these compounds in the presence of water and light, generally under anaerobic conditions and at room temperature, producing hydrogen and carbon dioxide as the main products [5].

Titanium dioxide (TiO_2_) remains one of the most widely studied photocatalysts because of its availability, chemical stability, low cost, and corrosion resistance [6,7,8,9]. Since the pioneering work of Fujishima and Honda, who first demonstrated the photoelectrochemical splitting of water on TiO_2_ electrodes under ultraviolet irradiation [10], this semiconductor has been widely studied in photocatalytic and photoelectrochemical applications. However, its practical application has intrinsic limitations that restrict its efficiency. On the one hand, TiO_2_ experiences rapid recombination of the electron-hole pairs generated after light excitation; it is estimated that more than 90% of photogenerated electrons recombine with holes on time scales of the order of nanoseconds (~10 ns), which drastically reduces the fraction of charge carriers available to participate in surface redox reactions. On the other hand, its wide band gap (~3.2 eV for anatase) limits light absorption mainly to the ultraviolet region of the solar spectrum, which represents only 5% of total solar irradiance [11,12]. Consequently, numerous strategies have been developed to improve TiO_2_ photocatalytic efficiency, including the formation of heterojunctions with other semiconductors [13,14,15], doping with transition metals [16,17,18] or non-metal elements [19], co-doping [20,21], surface modification with organic compounds or sensitizers [22,23], and control of morphology at the micro- and nanoscale [24,25,26], among others.

Among these strategies, micro- and nanoscale structural design has proven to be a particularly effective approach for improving light absorption, mass transport, and the separation of photogenerated charges [27,28]. In this context, biotemplated synthesis routes have attracted growing interest in recent years [29,30,31]. Natural biological structures exhibit hierarchical architectures that have been optimized over millions of years to maximize light capture and facilitate fluid and nutrient transport. Replicating these architectures in inorganic semiconductors enables the production of solids with interconnected porosity, high specific surface area, and complex microstructures. These features can improve light scattering and trapping and facilitate the diffusion of reactants and products during photocatalytic reactions [24,25,26,32,33,34].

From the various biotemplates studied, olive leaves represent a particularly attractive model. First, they are widely available as agro-industrial waste streams. The European Union is currently the world’s largest producer of olive oil, accounting for approximately 68% of global production. This production process generates large quantities of by-products, including olive pomace, olive oil mill waste, and olive leaves. It is estimated that around 10 million tons of waste are produced annually in the industrial sector. Approximately 3 million additional tons are generated in the agricultural sector, of which about 25% correspond to olive leaves [35]. In Spain, particularly in Andalusia, olive production is of fundamental economic and territorial importance. Andalusia has more than 1.6 million hectares dedicated to olive cultivation, an area that continues to increase due to the modernization of agricultural practices and the development of new irrigation techniques [36].

From a structural perspective, olive leaves have a complex vascular system and a multiscale hierarchical organization that includes transport channels, cavities, and porous structures that facilitate light capture. Replicating this natural architecture in TiO_2_-based materials preserves these structural features and generates solids with a highly organized microstructure. In previous work by our research group, we have demonstrated that preserving these hierarchical structures can enhance photocatalytic performance [24,25,26]. Consequently, these bioinspired architectures constitute a promising platform for further functionalization processes aimed at improving the utilization of charge carriers and optimizing the kinetics of surface reactions.

In addition to morphological control, the incorporation of metal cocatalysts is one of the most effective strategies for improving photocatalytic hydrogen production [37]. Noble metals deposited on TiO_2_ act as electron sinks, promoting charge separation by capturing photogenerated electrons and thus reducing recombination losses. This effect is due to the formation of a Schottky barrier at the metal–semiconductor interface, which allows the transfer of electrons from the conduction band of TiO_2_ to the metal, where they remain trapped, preventing their recombination with holes [38,39]. Among the various noble metals studied, platinum is widely recognized as one of the most effective cocatalysts for hydrogen evolution due to its optimal hydrogen adsorption energy and excellent catalytic properties [40,41]. Gold, in contrast, can not only facilitate electron capture but also introduce plasmonic effects associated with localized surface plasmon resonance (LSPR) under light irradiation. This phenomenon can increase visible light absorption and generate energetic electrons (“hot electrons”) [42].

For metal deposition, photodeposition is a particularly advantageous method for incorporating nanoparticles onto semiconductor surfaces. This method allows intimate contact between the metal and the semiconductor and favors the preferential nucleation of nanoparticles at sites where photogenerated electrons accumulate during irradiation. As a result, high metal dispersion and better particle size control are obtained, factors that directly influence the efficiency of cocatalysts and the dynamics of charge transfer at the metal–semiconductor interface [43].

Although previous studies have demonstrated the advantages of olive-leaf-biotemplated TiO_2_ and the beneficial role of noble metal cocatalysts in photocatalytic hydrogen production, these two approaches have mostly been investigated independently. In particular, the preservation of the hierarchical leaf architecture and the incorporation of noble metals have rarely been addressed in a combined and systematic manner. Moreover, comparative studies evaluating their joint effect under both UV and simulated solar irradiation remain limited.

In this context, the novelty of this work lies in the deliberate integration of hierarchical structuring via bio-templating with the photodeposition of noble metals on a single TiO_2_-based platform. Specifically, this study not only replicates the architecture of the olive leaf but also incorporates Pt and Au nanoparticles in a controlled manner, allowing for a direct and systematic comparison of their individual and synergistic effects under both UV irradiation and simulated solar irradiation. This approach provides new insights into how morphology and the cocatalyst jointly influence key processes such as charge separation, light harvesting, and overall photocatalytic hydrogen production, thereby surpassing the current state of the art, in which these factors have typically been considered in isolation.

## 2. Materials and Methods

### 2.1. Materials

Aeroxide® TiO_2_ P25 was supplied by Evonik (formerly Degussa). Glycerol ≥ 99.5% (Ref. G7893); titanium (III) chloride (Ref. 7705-07-9); titanium (IV) isopropoxide (Ref. 205273), chloroauric acid trihydrate ≥99.9% (Ref.520918); chloroplatinic acid hydrate ≥99.9% (Ref.520896) were purchased from Sigma Aldrich (Madrid, Spain). Propan-2-ol (isopropanol) was obtained from Merck (Ref. 33539-M) and 37 wt% hydrochloric acid (Ref. 141020) from Panreac. Milli-Q water was obtained from Millipore Corporation (Madrid, Spain).

### 2.2. Synthesis of Photocatalysts

The nano- and microstructures characteristic of olive leaf photosystems were reproduced through an ion-exchange process designed to mimic the natural degradation of chlorophyll, following a previously optimized synthetic procedure [24,25,26]. The method consists of three stages. In the first stage, an acid treatment was used to remove metal cations, mainly Mg^2+^, from porphyrins, which are replaced by H^+^, leading to the formation of yellow-brown pheophytins. For this initial stage, fresh olive leaves were washed with Milli-Q water and left to dry. Subsequently, the midrib was removed along with the petiole and apex, and the plant material was fragmented into pieces as small as possible. Next, 10 g of cut leaves were weighed and placed in a round-bottom flask with 150 mL of 5% HCl, which was stirred overnight in an inert atmosphere.

In the second stage, 30 mL of TiCl_3_ (0.061 mol) was added by injection through a septum. During this process, an ion exchange takes place in which the protons associated with the pheophytin group of chlorophyll are replaced by Ti^3+^, which become incorporated into the thylakoid nanostructure. In the third phase, the Ti^3+^ ions act as nucleation centers for the subsequent formation of a TiO_2_ structure that reproduces the architecture of the olive leaf. The leaves were filtered using a Büchner funnel, washed three times with 50 mL of Milli-Q water, and dried in a vacuum desiccator at 80 °C. Once completely dry, they were returned to the flask and suspended in 98 mL of isopropanol, keeping them in agitation throughout the night in order to remove residual water. The leaves were then filtered again, washed with isopropanol, and returned to a flask containing 98 mL of isopropanol and 9 mL of titanium isopropoxide. The suspension was stirred overnight. After this period, the mixture was refluxed at 95 °C for 6 h. Finally, the dry solid obtained was calcined in a muffle furnace at 550 °C for 6 h, using a heating ramp of 1 °C·min^−1^. The catalyst obtained was named AOL, corresponding to the acronym for “Artificial Olive Leaf.”

A 0.5% loading of Pt or Au was deposited onto the surface of the AOL material using a conventional photodeposition process. The appropriate volume of H_2_PtCl_6_ or HAuCl_4_, respectively, was added to a dispersion of the semiconductor in a 10% (*v*/*v*) methanol solution. The suspension was irradiated for 5 h using a 125 W medium-pressure Hg lamp (λmax = 365 nm, 564 mW·cm^−2^). Throughout the reaction, an Ar flow of 20 mL·min^−1^ was maintained, while the temperature was controlled at 25 °C by a water cooling system. After irradiation, the solids were recovered by filtration, washed, and dried at 80 °C overnight. The resulting samples were designated Pt/AOL and Au/AOL, depending on the photodeposited metal.

### 2.3. Characterization of Solids

Several characterization techniques were used to determine the morphology, structure, and composition of the catalysts synthesized by the biotemplating process.

The specific surface area and pore volume of the samples were determined from nitrogen adsorption–desorption measurements performed on a Quantachrome Instruments system (Autosorb-iQ-2-MP/XR Anton Paar Spain S.L.U., Madrid, Spain) at −196 °C. The solids were outgassed at 120 °C before the analysis. The surface area of the materials was determined by the BET method and the pore size distribution by the BJH method with the corrected form of the Kelvin equation. The equipment is available at the Chemical Institute for Energy and the Environment (IQUEMA) of the University of Cordoba.

X-ray diffraction patterns were recorded on a Bruker D8 Discover (Bruker Española S.A., Madrid, Spain) using a monochromatic CuKα1 source (λ = 1.54 Å), over a 2θ range of 10–80°, at a scan rate of 1.45° 2θ·min^−1^. The equipment is available at the Chemical Institute for Energy and the Environment (IQUEMA) of the University of Cordoba.

Diffuse reflectance spectra were obtained using an Agilent Cary 5000 UV-Vis-NIR spectrophotometer (Agilent, Santa Clara, CA, USA). The apparent band gap values were estimated using the Kubelka–Munk function and Tauc representation ([F(R)xE]^1/2^ versus photon energy).

X-ray photoelectron spectroscopy was performed on a Leibold-Heraeus LHS10 spectrometer (SPECS, Berlin, Germany), which had a chamber capable of operating at less than 2·10^−9^ Torr, equipped with an EA-200MCD hemispherical electron analyzer with an AlKα X-ray source (hν = 1486.6 eV) at 120 W, 30 mA. The C 1s signal at 284.6 eV was used as the reference. The equipment is available at the Central Service for Research Support (SCAI) of the University of Cordoba.

Scanning electron micrographs (SEM) were obtained using a JEOL JSM 7800F Prime microscope (JEOL Ltd., Tokyo, Japan). The equipment is available at the Central Service for Research Support (SCAI) of the University of Córdoba.

Transmission electron microscopy (TEM) images were acquired using a JEOL JEM 1400 transmission electron microscope. Samples were deposited onto 3 mm holey carbon-coated copper grids prior to analysis. Particle size distributions were determined using ImageJ 1.46r, a public-domain Java-based image processing software. All measurements were carried out at the Central Service for Research Support (SCAI) of the University of Córdoba. 

Elemental analysis of metal-containing samples was performed by inductively coupled plasma mass spectrometry (ICP-MS) using a PerkinElmer NexION X instrument after complete dissolution of the samples. Digestion was conducted in two steps: initially with a 1:1:1 H_2_O/H_2_SO_4_/HF mixture at 80 °C, followed by treatment with a 1:3 HNO_3_/HCl solution. The equipment is available at the Central Service for Research Support (SCAI) of the University of Córdoba.

### 2.4. Glycerol Photoreforming Reaction

The prepared catalysts were evaluated for hydrogen generation by photocatalytic reforming of a 10% (*v*/*v*) aqueous glycerol solution under two experimental configurations that differed in the irradiation source.

The ultraviolet irradiation tests were carried out in a double-walled cylindrical immersion reactor made of Pyrex (23 cm long and 5 cm internal diameter, with a total volume of 190 mL), equipped with a 125 W medium-pressure mercury lamp supplied by Photochemical Reactors Ltd. (Reading, UK). In each experiment, a suspension consisting of 65 mg of catalyst and 65 mL of 10% (*v*/*v*) glycerol solution was prepared. Throughout the reaction, argon was continuously passed at a flow rate of 5 mL·min^−1^ in order to maintain an inert atmosphere and carry away the gaseous products. The reaction started when the lamp was switched on. The generated hydrogen was analyzed online by gas chromatography with a thermal conductivity detector, model 7890A from Agilent Technologies (Santa Clara, CA, USA), equipped with a Supelco Carboxen ™ 1010 PLOT fused silica capillary column 30 m long, 0.32 mm ID, marketed by Sigma Aldrich (Darmstadt, Germany).

The reusability tests under UV irradiation were performed following the same experimental procedure described above. After each reaction cycle, the catalyst was recovered by centrifugation and subsequently reused in the next run under identical reaction conditions.

The experiments under simulated sunlight irradiation were carried out in an 8.5 mL cylindrical reactor operated in an argon atmosphere and irradiated with a Newport Corporation solar simulator equipped with a 150 W xenon lamp. The reaction system consisted of 4 mg of catalyst suspended in 4 mL of 10% (*v*/*v*) glycerol solution, with a headspace of 4.5 mL in the reactor for the accumulation of gaseous products. Gas samples were taken from the headspace using a precision analytical syringe with a pressure seal (1 mL, leak-tight up to 250 psi) from VICI Valco Instruments. The analysis was performed by GC-TCD (Agilent Technologies 7890A chromatograph using a Supelco Carboxen™ 1010 PLOT).

The amount of H_2_ produced was determined from a calibration curve obtained by controlled additions of pure hydrogen to the chromatograph, covering a concentration range between 0 and 20 µmol.

The incident irradiance power in the sample compartment, measured for wavelengths below 800 nm with an Ophir Optronics Starlite power meter, was 116 mW·cm^−2^ for the UV lamp and 106 mW·cm^−2^ for the solar simulator.

## 3. Results

### 3.1. Synthesis of Catalysts and Morphological Analysis by SEM

Olive leaves were used as a biological model for the design of TiO_2_-based photocatalytic materials, referred to here as artificial olive leaves (AOL). The biotemplated strategy aimed to reproduce both the external morphology and the internal microstructure of the natural leaf. This approach is based on the hierarchical anatomical organization of the leaf, whose upper mesophyll is formed by two or three layers of elongated and compact cells that constitute the palisade parenchyma, while the lower mesophyll corresponds to the spongy parenchyma, rich in intercellular spaces that increase the air fraction of the tissue [44]. This differentiated architecture favors efficient absorption and dispersion of solar radiation, optimizing the use of light.

In this study, three catalysts were synthesized. First, the AOL material was obtained following a biotemplated procedure in stages. Initially, the porphyrin ring was demetallized, removing the central Mg^2+^ and replacing it with a proton. In a subsequent step, Ti^3+^ cations were incorporated, generating precursor species that acted as nucleation centers for the growth of the TiO_2_ network through the controlled hydrolysis of Ti(iPrO)_4_. Finally, platinum or gold metal nanoparticles were photocatalytically deposited on the AOL support, resulting in the bifunctional Pt/AOL and Au/AOL systems, respectively.

**Figure 1:**
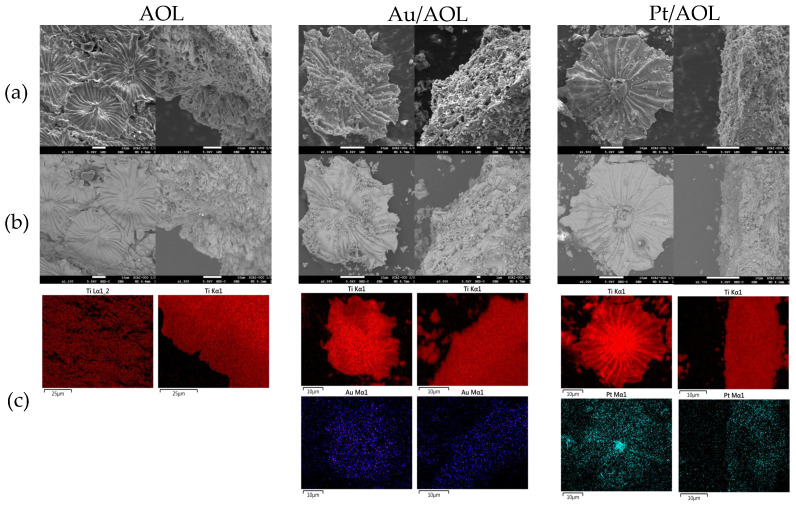
(**a**) SEM and (**b**) backscattered SEM images of AOL, Au/AOL and Pt/AOL. (**c**) Elemental mapping (SEM-EDS) of the samples indicating the distribution of Ti, Au or Pt.

To evaluate catalyst surface morphology and confirm faithful replication of the leaf structure, SEM analysis was performed. The results obtained are presented in Figure 1 and in Supplementary Figures S1–S3. The micrographs corresponding to the AOL solid show the presence of trichomes with a star-shaped morphology, characteristic of the reverse side of olive leaves. Observation of these structures confirms that the external architecture of the leaf has been successfully reproduced in the TiO_2_-based material. Likewise, cross-sectional analysis revealed preservation of the channeled pattern and spongy texture typical of the leaf, confirming faithful replication of both the surface morphology and internal structure.

In the case of catalysts obtained after the photodeposition process, SEM images show that the biotemplated structure is maintained after the incorporation of metals. However, a certain degree of fragmentation is observed, which can be attributed to mechanical agitation during the synthesis procedure. This suggests that milder stirring conditions or alternative reactor configurations could further improve the preservation of hierarchical AOL architecture. Despite this partial fragmentation, the distinctive morphological features of the leaf remain clearly identifiable. SEM-EDS analyses of the Pt/AOL and Au/AOL systems indicate homogeneous Pt and Au distribution over the TiO_2_ matrix replicating the olive leaf, with no evidence of metal agglomeration. Finally, the images obtained using backscattered electrons, where elements with higher atomic numbers appear brighter, do not show significant variations in contrast intensity. This behavior can be attributed to the small particle size of the deposited metals and their uniform dispersion on the substrate, which prevents the appearance of locally enriched metal areas that generate noticeable contrasts.

**Figure 2:**
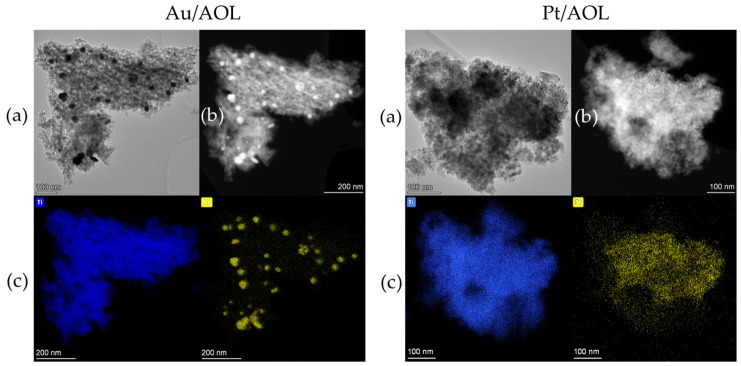
(**a**) TEM and (**b**) STEM images of Au/AOL and Pt/AOL. (**c**) Elemental mapping (STEM-EDS) of the samples indicating the distribution of Ti, Au or Pt.

To gain deeper insight into the nanoscale morphology and to more accurately assess both the size and distribution of the metallic phases on the support, transmission electron microscopy (TEM) was employed, complemented by STEM imaging and EDS analysis. The results for the Au/AOL and Pt/AOL catalysts are presented in Figure 2 and in Supplementary Figure S4.

The TEM micrographs (Figure 2a) reveal that both materials consist of an aggregated TiO_2_ matrix with a porous texture, consistent with the biotemplated structure observed by SEM. Darker nanoparticles can be clearly distinguished on this matrix, corresponding to the deposited metallic phases. These particles appear well anchored onto the TiO_2_ surface, with no evidence of large clusters of metallic particles. STEM images (Figure 2b), the metallic nanoparticles stand out with higher intensity, facilitating their identification. This assignment is further confirmed by EDS elemental mapping, which shows the spatial distribution of Ti, Au, and Pt. In both catalysts, titanium is homogeneously distributed, while Au and Pt signals appear as discrete spots dispersed throughout the structure, confirming the effective deposition of the metals.

A comparative analysis of the TEM micrographs highlights significant differences in the size and dispersion of the metallic nanoparticles. In the Au/AOL catalyst, gold nanoparticles exhibit an average size of approximately 24 nm and a relatively less homogeneous distribution. In contrast, in the Pt/AOL system, platinum nanoparticles are considerably smaller, with an average size of around 4 nm, and display a much more uniform dispersion over the catalyst surface. These differences suggest higher efficiency in the nucleation and stabilization of Pt species during the photodeposition process, leading to a finer and more homogeneous distribution, whereas Au tends to form larger particles with lower dispersion.

### 3.2. Structural Characterization of Catalyst

**Figure 3:**
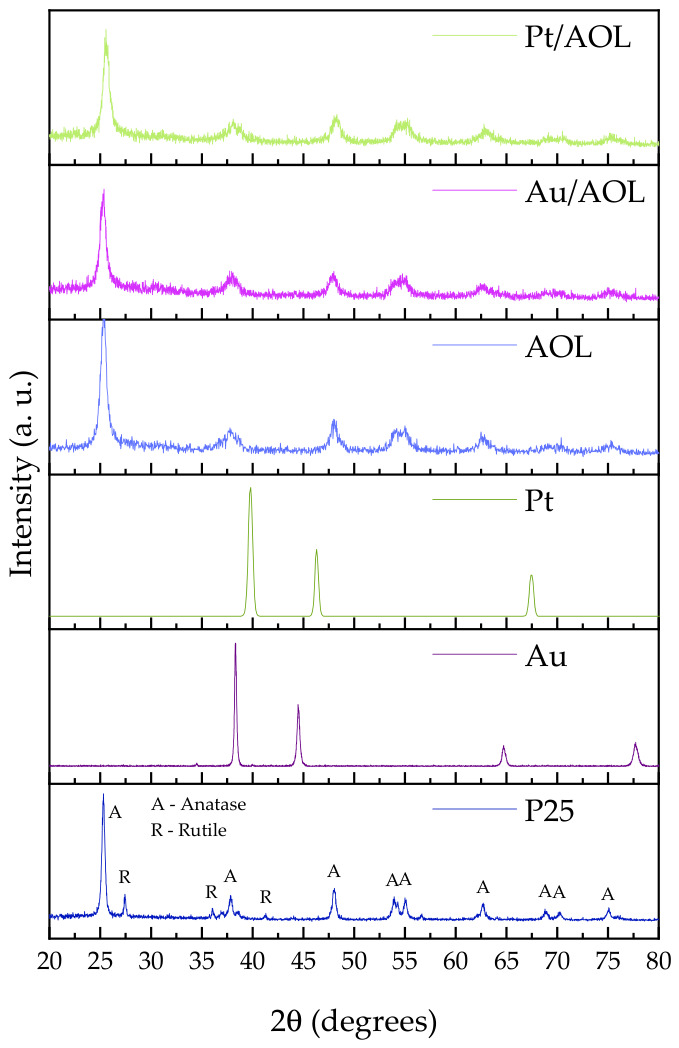
X-ray diffraction patterns of commercial titanium dioxide (Evonik P25), metallic Au and Pt reference patterns obtained from the RRUFF database, and the synthesized materials AOL, Au/AOL, and Pt/AOL.

The crystal structure of the catalysts was evaluated by X-ray diffraction using commercial Evonik P25 TiO_2_ and the Au and Pt reference patterns from the RRUFF database. (Figure 3). The P25 diffractogram shows narrow, intense peaks characteristic of high crystallinity, corresponding to an approximate composition of 80% anatase phase and 20% rutile phase. In particular, signals are identified at 2θ = 25.4, 37.9, 48.0, 54.0, 55.0, 62.7, 70.0, and 75.0°, attributable to the (101), (004), (200), (105), (211), (213), (116), and (215) planes of anatase [45,46]. Reflections at 27.5, 36.0, and 41.2° are also observed, associated with the (110), (101), and (111) planes of the rutile phase [46,47], confirming the biphasic nature of the commercial material.

Regarding the metallic reference patterns, gold shows well-defined peaks at 38.2, 44.6, 64.7, and 77.8°, corresponding to the (111), (200), (220), and (311) planes of its face-centered cubic (fcc) structure [48,49]. Metallic platinum, in turn, shows intense reflections around 39.8, 46.2, and 68.5°, assigned to the (111), (200), and (220) planes of its fcc crystal structure [50,51].

The diffractograms of the synthesized solids show greater background noise and less peak definition compared to commercial P25, suggesting a lower degree of crystallinity. However, the main reflections of the anatase phase are clearly distinguishable at 25.4, 37.9, 48.0, 54.0, 55.0, and 62.7°, associated with the (101), (004), (200), (105), (211), and (213) planes. Considering that AOL was calcined at 550 °C, a partial transformation of anatase into rutile would be expected. However, no characteristic rutile peaks are detected. This absence may be attributed to the relatively low crystallinity, the nanoscale size of the crystallites, and metal–support interactions, which can stabilize the anatase phase and hinder its transformation into rutile under these conditions. Furthermore, no significant shifts in peak position are observed between the three prepared catalysts, indicating that the incorporation of metals does not significantly alter the crystalline structure of the support.

The average crystallite size, estimated using the Scherrer equation, is similar for all solids, with an approximate value of 10 nm, with no significant differences between them. Finally, it should be noted that the characteristic reflections of metallic Au or Pt are not detected in Au/AOL and Pt/AOL catalysts, suggesting a high dispersion of the metallic phases or particle sizes below the detection limit of the technique.

**Figure 4:**
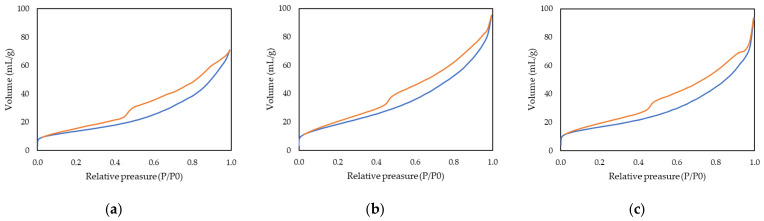
Nitrogen adsorption–desorption isotherm of (**a**) AOL, (**b**) Au/AOL and (**c**) Pt/AOL.

The N_2_ adsorption–desorption isotherms for all catalysts exhibit behavior characteristic of mesoporous materials (Figure 4). Specifically, type IV isotherms with an H3 hysteresis cycle were obtained, according to the IUPAC classification, which is indicative of the presence of mesopores generated by aggregates of laminar particles that can give rise to interconnected cavities [52]. This type of hysteresis is consistent with the hierarchical nature of the synthesized solids, whose architecture replicates both the external structure and the internal network of the olive leaf, forming interconnected channels and aggregates of flat particles.

Table 1 summarizes the BET specific surface area, average pore diameter, and total pore volume obtained from the N_2_ adsorption–desorption isotherms. After metal photodeposition, a slight increase in the specific surface area is observed for both catalysts, with the Au/AOL material showing the highest value. SEM observations indicate that the biotemplated structure is preserved after metal incorporation, although some degree of fragmentation is detected, likely due to the mechanical agitation during the synthesis procedure. This partial fragmentation could contribute to the slight increase in accessible surface area by exposing new surfaces or improving the accessibility of previously less accessible pores.

From a physicochemical perspective, the incorporation of small amounts of metal nanoparticles would not be expected to intrinsically increase the BET surface area. Therefore, the increase observed is more plausibly associated with structural effects induced during the photochemical treatment, such as slight particle disaggregation or improved exposure of internal surfaces.

Regarding the average pore diameter, a slight decrease is observed after gold deposition, whereas a small increase is detected for the platinum-containing catalyst. The decrease in the Au-containing material may be related to the preferential nucleation of nanoparticles inside the mesopores or near their entrances, which effectively reduces the average pore diameter without causing significant pore blockage. In contrast, the slight increase observed after Pt deposition may indicate a more external distribution of Pt nanoparticles or a mild restructuring of the pore network induced during photodeposition.

A similar trend is observed for the total pore volume, which slightly increases after the incorporation of both metals. Since the occupation of pores by metal nanoparticles would typically reduce the accessible volume, the observed increase suggests that pore obstruction is not the dominant effect. Instead, the photodeposition treatment may promote a certain degree of structural rearrangement or particle disaggregation, improving the accessibility and connectivity of the hierarchical mesoporous network.

Overall, these results confirm that the olive leaf biotemplating strategy produces stable hierarchical mesoporous structures. Moreover, the photodeposition of Au and Pt preserves replicated architecture while introducing subtle textural modifications that may be advantageous for photocatalytic applications.

**Figure 5:**
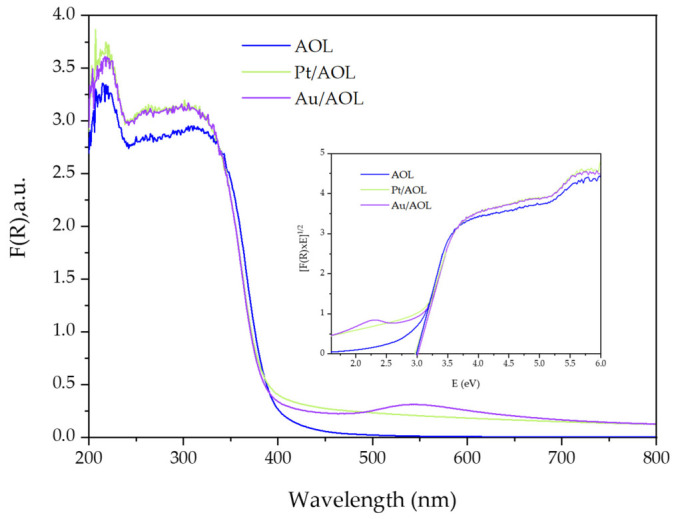
UV-Vis spectra AOL, Au/AOL and Pt/AOL and plot of the Kubelka–Munk function versus the energy of the absorbed light to determine the energy of the forbidden band.

To evaluate the optical properties of the synthesized materials, UV-Vis absorption spectra were recorded in the range 250–800 nm (Figure 5). In the case of the AOL solid, intense absorption is observed in the UV region (λ < 400 nm), characteristic of the ligand-to-metal charge transfer (LMCT) transition associated with O^2−^ → Ti^4+^ excitations typical of TiO_2_-based materials. However, the spectrum shows absorption extending toward longer wavelengths into the visible region (400–500 nm), indicating a red shift of the absorption edge relative to conventional TiO_2_. This contribution in the visible range may be associated with the presence of structural defects, particularly Ti^3+^ species, as well as the incorporation of residual elements from the olive leaf (C, Ca, P, K, S, Si, Fe, among others), which can introduce intermediate electronic levels within the band gap [53,54]. This red shift reduces the band gap energy value to 2.97 eV (Table 1), which is lower than the typical value for anatase TiO_2_ (~3.2 eV) [25,26]. This reduction can be explained by the generation of electronic levels associated with Ti^3+^ between the valence band and the conduction band, facilitating the absorption of radiation in the visible range [55].

For the Pt/AOL solid, there is a notable increase in absorption in the visible region (400–800 nm) compared to the unmodified AOL. Although the absorption edge also shows a red shift, no defined band or intense peak is detected in the visible region. The improvement in optical response can be attributed, first, to the ability of the deposited Pt species (Pt^0^, Pt(OH)_2_, and PtO_2_) to absorb visible radiation, as evidenced by UV-Vis diffuse reflectance spectra. Secondly, the dispersion of Pt nanoparticles on the TiO_2_ matrix can induce the formation of new electronic levels within the band gap, associated with metal–support interactions and the modification of the Fermi level at the Pt/TiO_2_ interface [56,57]. These effects favor absorption in the visible range without significantly altering the band gap energy, which remains practically constant (2.96 eV).

In the Au/AOL material, a substantial improvement in absorption in the 400–800 nm region is also observed compared to AOL. Unlike the Pt system, in this case a well-defined absorption peak is identified around 550 nm, attributable to the localized surface plasmon resonance (LSPR) characteristic of Au nanoparticles [42,58]. The absence of a second band in the infrared region indicates that the nanoparticles predominantly have a spherical morphology, since anisotropic particles (e.g., nanorods) would show additional plasmonic modes at longer wavelengths [59]. As in the case of Pt/AOL, modification with Au does not produce significant variations in the band gap energy, which remains at a value of 2.98 eV.

It should be noted that the application of Tauc plots to metal–semiconductor composite systems have important limitations. The presence of metallic nanoparticles can introduce defect states, localized optical transitions, and interfacial charge-transfer processes that modify the absorption profile [60]. Therefore, the band gap values obtained in this work should be considered as apparent optical band gaps rather than intrinsic electronic band gap energies.

**Figure 6:**
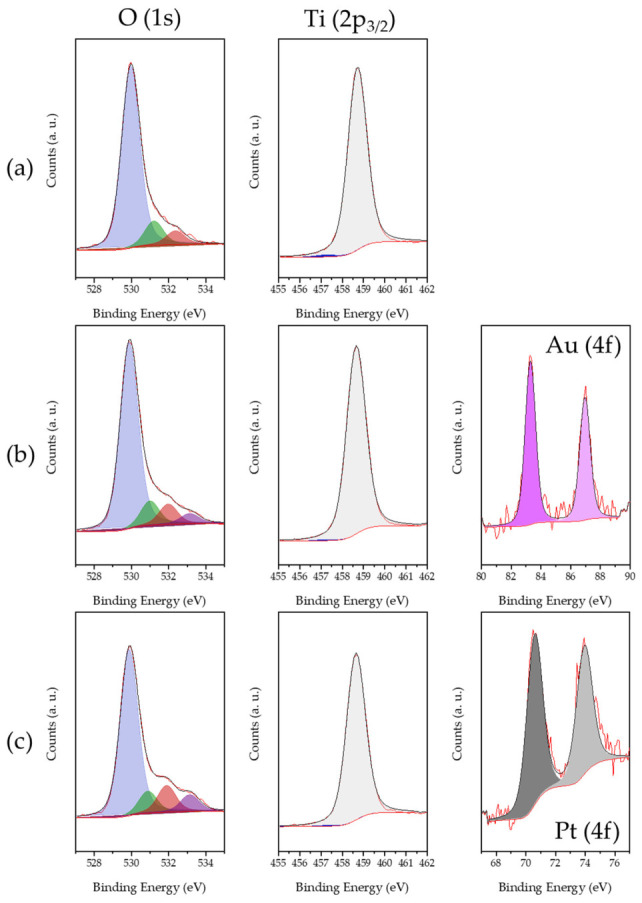
XPS spectra of O (1s), Ti (2p_3/2_), and/or Au (4f) and/or Pt (4f) for (**a**) AOL, (**b**) Au/AOL, and (**c**) Pt/AOL.

The surface chemical environment of the catalysts was analyzed using XPS. Figure 6 shows the XPS spectra corresponding to the synthesized solids. The Ti 2p_3_/_2_ signal can be decomposed into two contributions located at approximately 458.5 eV and 457.0 eV, attributable to the Ti^4+^ and Ti^3+^ species, respectively [61,62,63]. The Ti^3+^ fraction represents approximately 5.6% of the total titanium in the AOL solid, while in the Au/AOL and Pt/AOL catalysts this proportion decreases to 2.9% and 2.8%, respectively. These results indicate that biotemplating with olive leaf, as previously demonstrated, favors the generation of oxygen vacancies in the TiO_2_ structure, causing structural defects associated with the presence of Ti^3+^ species [25].

The O 1s spectrum of AOL solid shows three contributions centered approximately at 530.0, 531.2, and 532.4 eV, assigned in the literature to lattice oxygen (O^2−^), surface hydroxyl groups (–OH), and chemisorbed molecular oxygen (O_2_), respectively. In particular, the contribution located around ~532.4 eV is usually related to surface defects, such as oxygen vacancies, which favor O_2_ adsorption [64,65]. This result confirms the presence of surface defects in the material, consistent with the detection of Ti^3+^ species in the Ti 2p analysis.

For the Au/AOL and Pt/AOL catalysts, the O 1s spectra show the same main contributions, although slightly shifted towards lower binding energies, at approximately 529.9, 531.0, and 532.0 eV. This shift is a common phenomenon after the deposition of noble metals on TiO_2_ and is generally interpreted as evidence of metal–support interaction. Furthermore, an additional signal appears at ~533.2 eV in both catalysts, which in the literature is associated with adsorbed water or surface oxygenated species such as alcohols, carbonyls, or isolated esters, and can therefore be attributed to species adsorbed during the photodeposition process [66,67,68].

The high-resolution Au 4f XPS spectra of the Au/AOL catalyst fit two peaks at 83.3 and 87.0 eV, corresponding to Au^0^ 4f_7_/_2_ and Au^0^ 4f_5_/_2_, respectively [69]. These values appear slightly shifted from the typical binding energies of metallic Au (84.0 and 87.7 eV). This shift can be attributed to an electronic redistribution at the Au–TiO_2_ interface, caused by differences in the work functions and Fermi levels of both materials. Likewise, the possible electron transfer from the oxygen vacancies in TiO_2_ to the Au nanoparticles increases the electron density of the metal, which explains the observed decrease in binding energies [70].

Similarly, the Pt 4f spectrum of the Pt/AOL catalyst show two peaks centered at 70.6 and 74.0 eV, assigned to Pt^0^ 4f_7_/_2_ and Pt^0^ 4f_5_/_2_, respectively. These values are slightly below the characteristic binding energies of metallic Pt (~71.3 and 74.6 eV) [71,72,73]. Similar to the case of gold, this shift is related to electronic redistribution at the Pt–TiO_2_ interface and to the possible transfer of electrons from oxygen vacancies in the support to Pt particles, which increases the electronic density of the metal and reduces its binding energies.

A further point worth highlighting is that, unlike conventional methods for depositing metals on TiO_2_—such as impregnation or deposition–precipitation, which typically yield metal species predominantly in oxidized forms—photodeposition enables the direct formation of metals in their zero-valent state. This has been confirmed by XPS analysis and is well supported in the literature [74]. As a result, additional post-synthesis reduction treatments are unnecessary, and the induction period often associated with the in situ reduction of metal species during the reaction is effectively avoided.

Semi-quantitative XPS analysis, which probes only the outer few nanometers of the material, revealed surface Au and Pt contents of 0.36% and 0.41% for the Au/AOL and Pt/AOL catalysts, respectively. Based on these values, photodeposition yields of 72% for Au and 82% for Pt were initially estimated.

However, since XPS penetrates only a few nanometers into the solid, inductively coupled plasma mass spectrometry was used to accurately determine the total metal content in the bulk material. ICP-MS analysis confirmed Au and Pt loadings of 0.49% and 0.47%, respectively. These values correspond to overall photodeposition yields of 98% for Au and 94% for Pt, indicating an almost complete incorporation of the metals throughout the entire solid.

### 3.3. Photocatalytic Experiments

**Figure 7:**
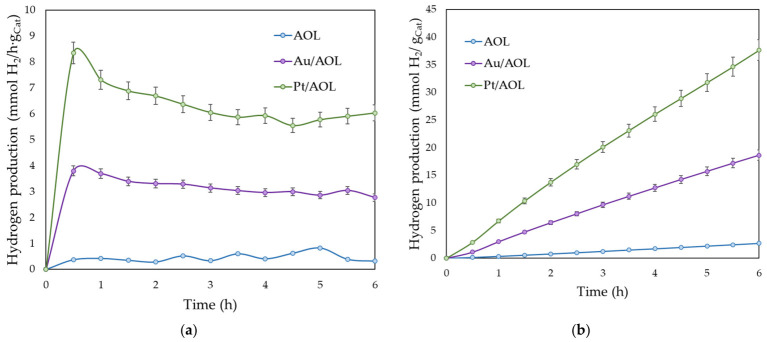
(**a**) the hydrogen production rate and (**b**) the accumulated hydrogen production from the photoreforming of aqueous glycerol solutions (10% *v*/*v*) under UV irradiation with the catalysts synthesized in this study. All experiments were performed in duplicate, and the deviation between runs was below 5%.

The photocatalytic efficiency of the synthesized materials was evaluated through glycerol photoreforming under UV and simulated solar irradiation. Figure 7 shows the results corresponding to hydrogen photoproduction under UV light. Figure 7a shows the reaction profiles of the different catalysts, where a significant improvement in photoactivity is observed after the incorporation of metals. The AOL solid exhibits a nearly constant production rate, with an average rate of 0.45 mmol H_2_·h^−1^·g_cat_^−1^. In contrast, metal-containing catalysts exhibit different behavior, showing maximum hydrogen production around 30 min of reaction, reaching values of 3.8 and 8.3 mmol H_2_·h^−1^·g_cat_^−1^ for Au/AOL and Pt/AOL, respectively. Subsequently, the rate decreases until it stabilizes at values close to 3 and 6 mmol H_2_·h^−1^·g_cat_^−1^ for Au/AOL and Pt/AOL, respectively.

Cumulative hydrogen production (Figure 7b) increased continuously over the course of the reaction. However, after the first 30 min, a slight deviation from linearity is observed, in accordance with the decrease in instantaneous velocity described above. After 6 h of UV irradiation, the cumulative production of H_2_ reaches values of 2.7, 18.6, and 37.7 mmol·g_cat_^−1^ for AOL, Au/AOL, and Pt/AOL, respectively. These results indicate that gold incorporation increased hydrogen production approximately sevenfold relative to the support, whereas platinum increased it by about fourteenfold, also doubling the activity observed for Au/AOL.

The improvement observed can be attributed to the ability of noble metals to form a Schottky barrier at the metal–semiconductor interface. When the metal comes into contact with the semiconductor, their Fermi levels are aligned, producing an electron transfer that generates a space charge region and an energy barrier at the interface. As a result, photoexcited electrons in the semiconductor migrate toward the metal, which acts as an electron sink, reducing electron–hole recombination. This process improves charge separation, increases the lifetime of electrons, and promotes the reduction reactions responsible for hydrogen generation. Although both gold and platinum can form this Schottky barrier, its efficiency depends on the electronic affinity of the semiconductor, which is identical in this case, and on the work function of the metal [38,39]. A higher work function implies a higher barrier height, which hinders the return of electrons from the metal to the semiconductor and favors their accumulation in the metal. Because the work function of platinum is approximately 5.6 eV [39], whereas that of gold is close to 4.5 eV [75], the higher Schottky barrier may help explain the higher photocatalytic activity of Pt/AOL. Furthermore, theoretical studies indicate that the interaction between hydrogen and metal surfaces is strongly dependent on the nature of the metal. Platinum exhibits significantly stronger hydrogen adsorption than gold [40,41]. This behavior has been associated with the superior catalytic efficiency of Pt-group metals in hydrogen-related reactions, as a more favorable metal–hydrogen interaction can facilitate the reduction steps involved in hydrogen evolution. Therefore, in addition to the electronic effects associated with Schottky barrier formation, the stronger interaction between platinum and hydrogen species may also contribute to the enhanced photocatalytic performance observed for the Pt/AOL system.

**Figure 8:**
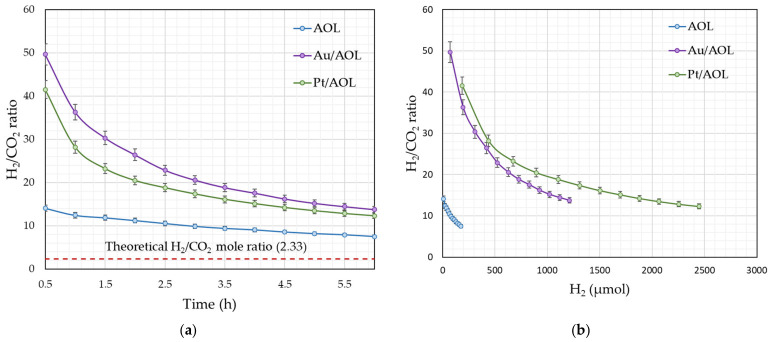
(**a**) Evolution of the H_2_/CO_2_ ratio during the reaction of the synthesized catalysts and (**b**) Evolution of the H_2_/CO_2_ ratio as a function of hydrogen produced. All experiments were performed in duplicate, and the deviation between runs was below 5%.

The evolution of the H_2_/CO_2_ ratio during glycerol photoreforming (Figure 8a) shows a progressive decrease over the reaction time. However, after 6 h none of the catalysts reaches the theoretical stoichiometric ratio of 2.3. This result confirms that glycerol mineralization is not complete under the conditions studied and that a significant fraction of the carbon remains in the form of organic intermediates in the liquid phase.

This phenomenon can be explained by the glycerol photoreforming mechanism proposed in the literature. The reaction begins with the activation and dehydrogenation of glycerol, mainly generating three-carbon intermediates such as glyceraldehyde and dihydroxyacetone. These molecules act as precursors for subsequent transformations in which carbon chain fragmentation processes occur. As a result, compounds with fewer carbons are formed, including C_2_ species such as glycolaldehyde or glycolic acid, and C_1_ products such as formaldehyde and formic acid, which can continue to oxidize to CO_2_ [76]. These transformations can begin through the oxidation of different centers of glycerol. When the reaction occurs at the terminal carbon (C1), glyceraldehyde is formed (route 1), while the oxidation of the secondary carbon (C2) leads to dihydroxyacetone (route 2). In addition to these routes, an alternative mechanism has also been described in which oxidative cleavage of the C–C bond occurs at an early stage, directly generating smaller molecules such as glycolaldehyde, formaldehyde, and hydrogen (Route 3) [77].

Analysis of the H_2_/CO_2_ ratio allows us to infer which of these routes might predominate in each catalyst. The AOL support exhibits significantly lower initial H_2_/CO_2_ values than the metal-loaded catalysts, suggesting a greater contribution of early C–C bond cleavage (route 3), which promotes faster CO_2_ formation at the initial stages. In contrast, Au/AOL and Pt/AOL display higher initial H_2_/CO_2_ ratios, consistent with enhanced glycerol dehydrogenation rates when glycerol concentration is highest, followed by subsequent C–C bond cleavage. This behavior points to a predominance of routes 1 and 2 in the presence of noble metals.

In principle, these apparent differences in reaction route between the metal-loaded catalysts and AOL could reflect the lower intrinsic activity of the support. This lower activity could bias the mechanistic interpretation and make the catalysts appear to follow different pathways even if they all proceed through the same overall scheme. To distinguish between these two possibilities, Figure 8b shows the H_2_/CO_2_ ratio as a function of the amount of hydrogen produced.

If all catalysts followed the same mechanism, similar H_2_/CO_2_ values would be expected at comparable H_2_ production levels. However, Au/AOL and Pt/AOL exhibit nearly overlapping profiles, suggesting a common mechanistic pathway dominated by initial dehydrogenation steps. In contrast, the AOL support shows consistently lower H_2_/CO_2_ ratios even at similar hydrogen production levels, indicating that its behavior is not solely due to lower activity, but rather to a different sequence of elementary steps, with a stronger contribution from early C–C bond cleavage. However, this interpretation remains indirect because no liquid-phase analysis (e.g., HPLC or GC-MS) was performed to identify reaction intermediates experimentally. Therefore, the proposed reaction scheme should be considered as a plausible mechanistic interpretation rather than definitive proof.

Overall, these results indicate that, beyond enhancing photocatalytic activity, the incorporation of noble metals shifts the glycerol photoreforming pathway toward mechanisms in which dehydrogenation precedes C–C bond cleavage.

To evaluate the stability and reusability of the catalysts, the material with the highest hydrogen production, Pt/AOL, was selected and subjected to five consecutive photocatalytic cycles. As shown in Figure S5, the general hydrogen evolution profile remains consistent throughout all cycles, with an initial maximum production rate reached within the first ~30 min, followed by a gradual decrease until a steady-state regime is established. This behavior suggests that the fundamental reaction mechanism and photoresponse of the metal-loaded solid are preserved upon reuse.

**Figure 9:**
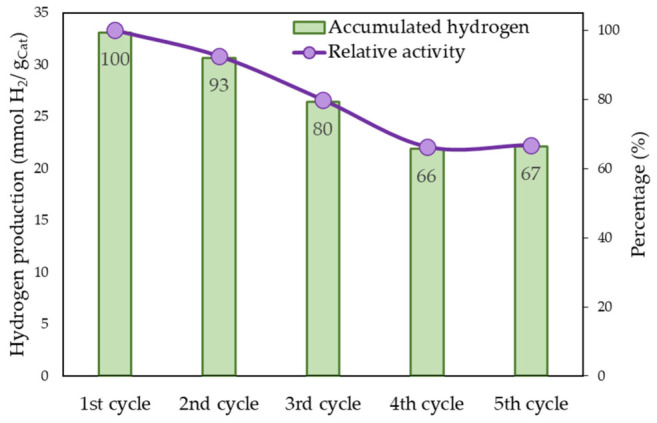
Cumulative hydrogen production after 6 h of reaction and relative activity (%) for each cycle compared to the first use.

However, a progressive loss of catalytic activity is observed after each glycerol photoreforming cycle. Focusing on the accumulated hydrogen production after 6 h (Figure 9), a clear decline in performance is evident with successive cycles. Specifically, hydrogen production decreases to 93% of the initial value in the second cycle and to 80% in the third cycle. From the fourth cycle onward, the activity stabilizes at approximately 70% of the initial performance, corresponding to an average hydrogen production of around 22 mmol·g_cat_^−1^.

This gradual deactivation may be attributed to factors such as partial blockage of active sites by reaction intermediates, slight Pt nanoparticle agglomeration, or surface modifications under reaction conditions. These results indicate that although the Pt/AOL catalyst undergoes partial deactivation during reuse, it reaches a relatively stable performance after the fourth cycle, maintaining about 70% of its initial hydrogen production capacity.

The deposition of noble metals on TiO_2_ not only promotes charge separation and reduces e^−^/h^+^ recombination, as observed under UV irradiation, but also modifies the optical properties of the semiconductor. In particular, as shown by the UV–Vis spectra, the incorporation of metals induces an extension of TiO_2_ absorption towards the visible region of the spectrum, potentially allowing a greater fraction of solar radiation to be used.

**Figure 10:**
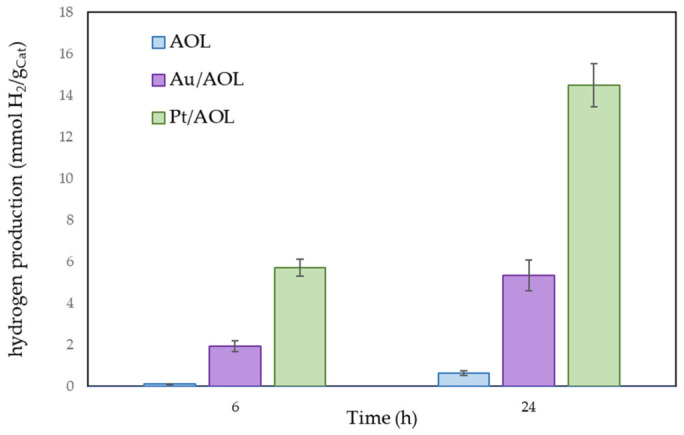
Hydrogen photoproduction by AOL, Au/AOL, and Pt/AOL under simulated sunlight for 6 and 24 h. All experiments were performed in duplicate, and the deviation between runs was below 5%.

In order to evaluate this effect, glycerol photoreforming experiments were carried out under simulated solar irradiation, the results of which are shown in Figure 10. As expected, the AOL solid, based only on TiO_2_, shows the lowest photocatalytic activity. This behavior is due to the fact that TiO_2_ absorbs mainly in the UV region of the spectrum. However, mimicking the structure of the olive leaf contributes to slightly improving light capture and partially shifting absorption towards the visible region. Despite this, the simulated solar radiation used contains only about 5% UV light, which significantly limits the generation of electron-hole pairs in this material and, consequently, its photocatalytic activity.

Similar to what was observed under UV irradiation, the Pt/AOL catalyst exhibits the highest photocatalytic activity. After 24 h of reaction, Au/AOL achieved hydrogen production approximately eightfold higher than AOL. Pt/AOL showed an even larger improvement, with production around twenty-threefold higher than the support and almost threefold higher than Au/AOL. It should be noted that these differences are more pronounced than those observed under UV irradiation. This can be explained by the fact that, under simulated sunlight, the improvement in activity is not only associated with the greater efficiency in charge separation induced by metals, but also with the extension of optical absorption towards the visible region.

Likewise, the differences in activity between metals are more evident under these conditions. As can be seen in the absorption spectra, the Pt/AOL solid exhibits more intense absorption across the entire spectrum, which facilitates electronic excitation under lower-energy radiation. Taken together, this could help explain the higher photocatalytic efficiency observed in the platinum-based system under simulated solar radiation.

In summary, the improved photocatalytic performance arises from different contributions depending on the irradiation conditions. Under UV irradiation, the enhancement is primarily governed by the formation of a Schottky barrier at the metal–semiconductor interface, which facilitates electron trapping and suppresses charge recombination. This effect is common to both Pt and Au. Under simulated solar irradiation, however, additional effects become relevant. In the case of Au, the presence of a localized surface plasmon resonance (LSPR) band centered at ~550 nm enables enhanced visible-light absorption and may contribute to photocatalytic activity through hot electron injection into the TiO_2_ conduction band. Nevertheless, despite this potential plasmonic contribution, the Pt/AOL catalyst exhibits higher activity than Au/AOL. This suggests that charge separation efficiency and the intrinsic catalytic properties of Pt play a more dominant role than plasmonic effects under the conditions studied.

Therefore, although plasmonic effects may contribute to the performance of Au/AOL under visible light, the overall photocatalytic enhancement is mainly driven by Schottky barrier formation and strong metal–support interactions, particularly in the case of Pt.

To compare the catalysts synthesized in this work with those reported in the literature, photocatalytic activity should be normalized to TiO_2_ as a reference. The literature uses a wide variety of experimental conditions (such as different irradiation sources, sacrificial agents, or light intensities), which makes it difficult to directly compare the absolute values of H_2_ production. Therefore, the most appropriate criterion is to use the improvement factor, defined as the ratio between the hydrogen production obtained with the modified catalyst and that corresponding to pure TiO_2_ under the same experimental conditions. This approach minimizes the variability associated with different experimental protocols.

The beneficial effect of olive leaf biotemplated on TiO_2_-based materials has been previously demonstrated by our group. In these studies, it was observed that AOL solid exhibited approximately twice the activity of P25 under UV irradiation and around 1.5 times greater activity under simulated sunlight [25,26].

For this reason, in order to establish a consistent comparison with the results available in the literature, values reported for metal systems supported on TiO_2_ have been considered, which are summarized in Table 2. The data compiled in this table show that the biotemplated support derived from olive leaf (AOL) significantly increases photocatalytic performance. In particular, Au/AOL and Pt/AOL catalysts show clearly superior improvement factors compared to those observed for conventional M/TiO_2_ catalysts evaluated under equivalent conditions.

Under ultraviolet irradiation, the Au/AOL catalyst achieves an enhancement factor of 6.9, a value that exceeds the ranges typically reported for conventional TiO_2_-supported Au systems, which are between 1.4 and 5.8 [78,79,80]. This trend is even more pronounced in the case of Pt/AOL, which shows an improvement factor of 14.0 compared to the reference TiO_2_. This value is three times higher than the increase observed in some previously described catalysts [81], or remains within the same order of magnitude as other highly efficient systems reported in the literature [82,83], confirming the high efficiency of the biotemplated support under UV irradiation.

However, the most notable results were obtained under simulated sunlight. Under these conditions, the Au/AOL system has an enhancement factor of 8.4, exceeding the values between 4.3 and 6.3 described for Au/TiO_2_ catalysts with comparable or even higher metal loads [84,85,86].

The most relevant result of this study corresponds to the Pt/AOL catalyst under sunlight, which exhibits an improvement factor of 22.8. This value is significantly higher than those reported for conventional Pt/TiO_2_ systems consulted in the literature, ranging between 4.8 and 7.1 [84,87,88]. This more than threefold increase relative to previously reported values indicates that the combination of biotemplated structuring and noble metal photodeposition is an effective strategy for enhancing the photocatalytic activity of TiO_2_-based materials.

The results demonstrate that the combination of biotemplated hierarchical structuring and noble metal photodeposition leads to performance improvements that exceed those typically reported for conventional TiO_2_-based systems. This highlights the added value of integrating morphology control and cocatalyst engineering within a single material design strategy.

## 4. Conclusions

Biotemplated synthesis using olive leaves successfully reproduced both the external morphology and the hierarchical internal structure of the natural leaf, as confirmed by SEM analysis. Although some fragmentation occurred during the photodeposition process due to mechanical agitation, the characteristic leaf-like architecture remained clearly identifiable. TEM analysis reveals clear differences in nanoparticle characteristics: Au/AOL shows larger Au particles (~24 nm) with less uniform dispersion and some aggregation, whereas Pt/AOL exhibits much smaller Pt nanoparticles (~4 nm) with a highly homogeneous distribution across the surface. XRD analysis revealed a relatively low crystallinity with anatase as the only detected TiO_2_ phase, and the absence of diffraction peaks for Au or Pt suggests high dispersion and small particle size of the deposited metals. Nitrogen adsorption–desorption measurements further confirmed that the catalysts retain a stable hierarchical mesoporous structure that replicates the olive leaf architecture, with the photodeposition of noble metals preserving the porous network and slightly improving pore accessibility without significant blockage. Optical characterization showed that the biotemplated synthesis reduced the TiO_2_ band gap to approximately 2.97 eV, likely due to structural defects and residual elements introduced during the templating process, shifting light absorption toward the visible region. In addition, Pt enhanced the optical response through metal–support electronic interactions, whereas Au introduced an additional absorption band at around 550 nm associated with localized surface plasmon resonance (LSPR). XPS analysis confirmed the formation of oxygen vacancies and Ti^3+^ species in the TiO_2_ lattice, which promote electron transfer to the noble metal nanoparticles and generate strong metal–support interactions, as evidenced by the shift of binding energies toward lower values. ICP-MS confirmed bulk metal loadings of 0.49% Au and 0.47% Pt, corresponding to high photodeposition yields (98% and 94%, respectively) and indicating nearly complete incorporation of both metals into the solid.

As a result, noble metal incorporation significantly enhanced hydrogen production during glycerol photoreforming. For the Pt-modified catalyst, increases of up to 14-fold under UV irradiation and 23-fold under simulated solar radiation were achieved. This improvement is mainly attributed to the formation of Schottky barriers that facilitate electron trapping and reduce charge recombination, with Pt exhibiting superior performance due to its higher work function. Finally, the analysis of the H_2_/CO_2_ ratio suggests that the AOL support tends to favor degradation pathways involving early C–C bond cleavage, whereas the presence of Au or Pt promotes initial glycerol dehydrogenation steps, delaying CO_2_ formation and indicating that noble metal incorporation not only enhances hydrogen production but also modifies the dominant reaction route during glycerol photoreforming. In terms of stability, the Pt/AOL catalyst maintains its photocatalytic behavior upon reuse but exhibits a gradual deactivation, with hydrogen production decreasing to ~70% after three cycles before stabilizing.

## Figures and Tables

**Table 1:** BET surface area, average pore size, pore volume, and band gap of the synthesized catalysts.

Catalyst	BET Surface Area (m^2^/g)	Average Pore Size (Å)	Pore Volume (mL·g^−1^)	Band Gap (eV)
AOL	50	44.3	0.11	2.97
Au/AOL	71	41.6	0.15	2.98
Pt/AOL	60	48.4	0.15	2.96

**Table 2:** Comparison of hydrogen photoproduction by synthesized catalysts with other catalysts described in the literature.

Catalyst	Sacrificial Agent	Light Source	Hydrogen Production (TiO_2_) (mmol·g_cat_^−1^)	Hydrogen Production (Catalyst) (mmol·g_cat_^−1^)	Improvement Factor	Reference
0.5%-Au/AOL	Glycerol at 10%	UV	2.69	18.65	6.9	This work
0.25%-Au/TiO2	Methanol at 25%	UV	1.09	3.64	3.3	[78]
2%-Au/TiO2	Methanol at 50%	UV	12.00	70.00	5.8	[79]
0.5%-Au/TiO2	Methanol at 15%	UV	3.31	4.68	1.4	[80]
0.5 %-Pt/AOL	Glycerol at 10%	UV	2.69	37.66	14.0	This work
1%-Pt/TiO2	Methanol at 6.7%	UV	10.80	32.40	3.0	[81]
0.5%-Pt/TiO2	Glycerol at 10%	UV	11.40	148.20	13.0	[82]
0.5%-Pt/AOL	Glycerol at 10%	UV	0.8	8	10	[83]
0.5%-Au/AOL	Glycerol at 10%	Solar	0.64	5.33	8.4	This work
1%-Au/TiO2	Methanol at 10%	Solar	7.00	30.00	4.3	[84]
1%-Au/TiO2	Methanol at 10%	Solar	0.62	3.92	6.3	[85]
1%-Au/TiO2	Ammonia borane at 12%	Solar	0.85	4.40	5.2	[86]
0.5%-Pt/AOL	Glycerol at 10%	Solar	0.64	14.50	22.8	This work
2%-Pt/TiO2	Methanol at 12.5%	Solar	1.30	6.30	4.8	[87]
1%-Pt/TiO2	Methanol at 25%	Solar	12.64	89.60	7.1	[88]
1%-Pt/TiO2	Methanol at 10%	Solar	7.00	42.00	6.0	[84]

## Data Availability

The raw data supporting the conclusions of this article will be made available by the authors on request.

## Supplementary Materials

The following supporting information can be downloaded at: https://www.mdpi.com/article/10.3390/biomimetics11050300/s1, Supplementary Figure S1: (a) SEM and (b) Backscattered SEM images of AOL (c) Elemental mapping (SEM-EDX) of the samples indicating the distribution of Ti. Supplementary Figure S2: (a) SEM and (b) Backscattered SEM images of Au/AOL. (c) Elemental mapping (SEM-EDX) of the samples indicating the distribution of Ti and Au. Supplementary Figure S3: (a) SEM and (b) Backscattered SEM images of Pt/AOL. (c) Elemental mapping (SEM-EDX) of the samples indicating the distribution of Ti and Pt. Supplementary Figure S4. (a) TEM and (b) STEM images of Au/AOL and Pt/AOL. (c) Elemental mapping (STEM-EDS) of the samples indicating the distribution of Ti, Au or Pt. Supplementary Figure S5. Hydrogen production rate over five consecutive cycles using the Pt/AOL catalyst under UV irradiation. All experiments were performed in duplicate, and the deviation between runs was below 5%.
