# Supplementary material for: Biotemplated Artificial Olive Leaf-Structured TiO2 Decorated with Pt and Au for Enhanced Photocatalytic Hydrogen Production
Source: Biomimetics (Basel). 2026 Apr 26;11(5):300. doi: 10.3390/biomimetics11050300 (PMC13204767; doi:10.3390/biomimetics11050300)
Supplement: Supplementary file 1 [file biomimetics-11-00300-s001.zip › biomimetics-4238629-supplementary.pdf]

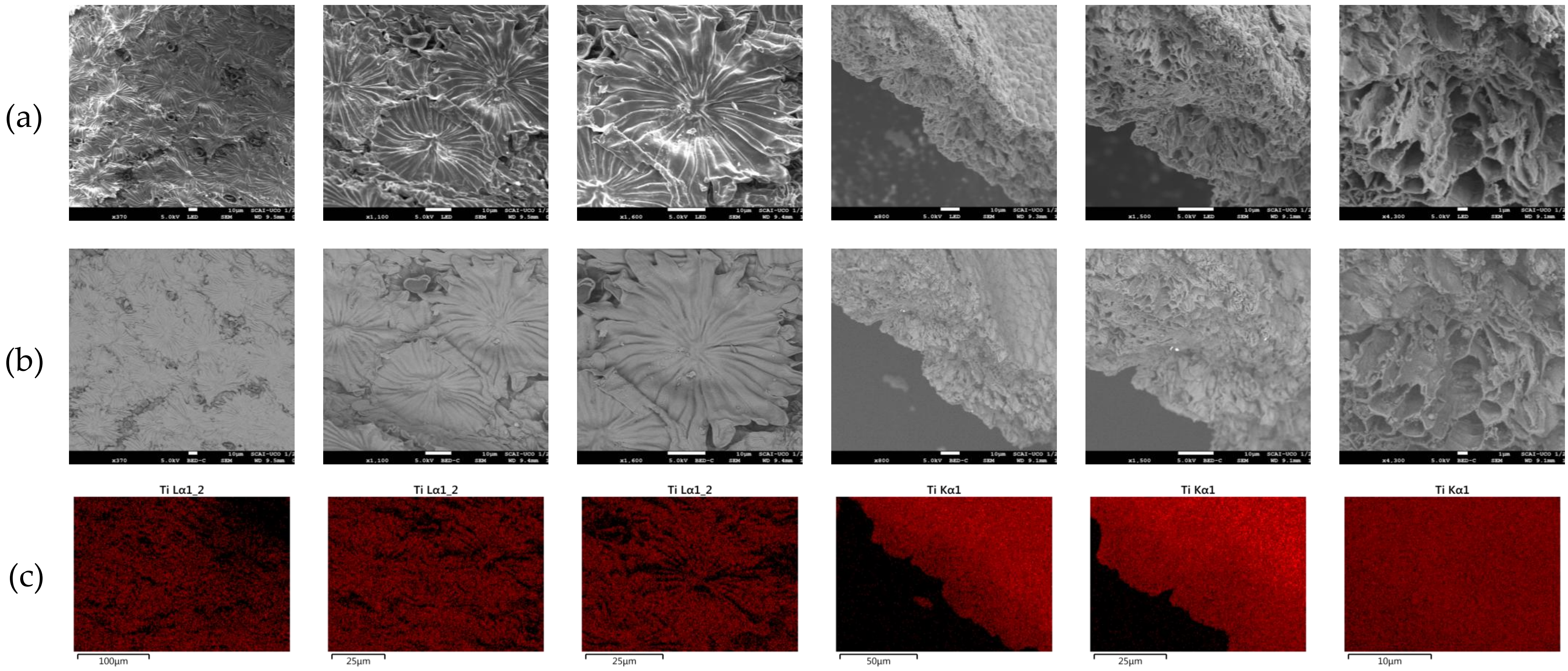

Figure S1. (a) SEM and (b) Backscattered SEM images of AOL (c) Elemental mapping (SEM-EDX) of the samples indicating the distribution of Ti.

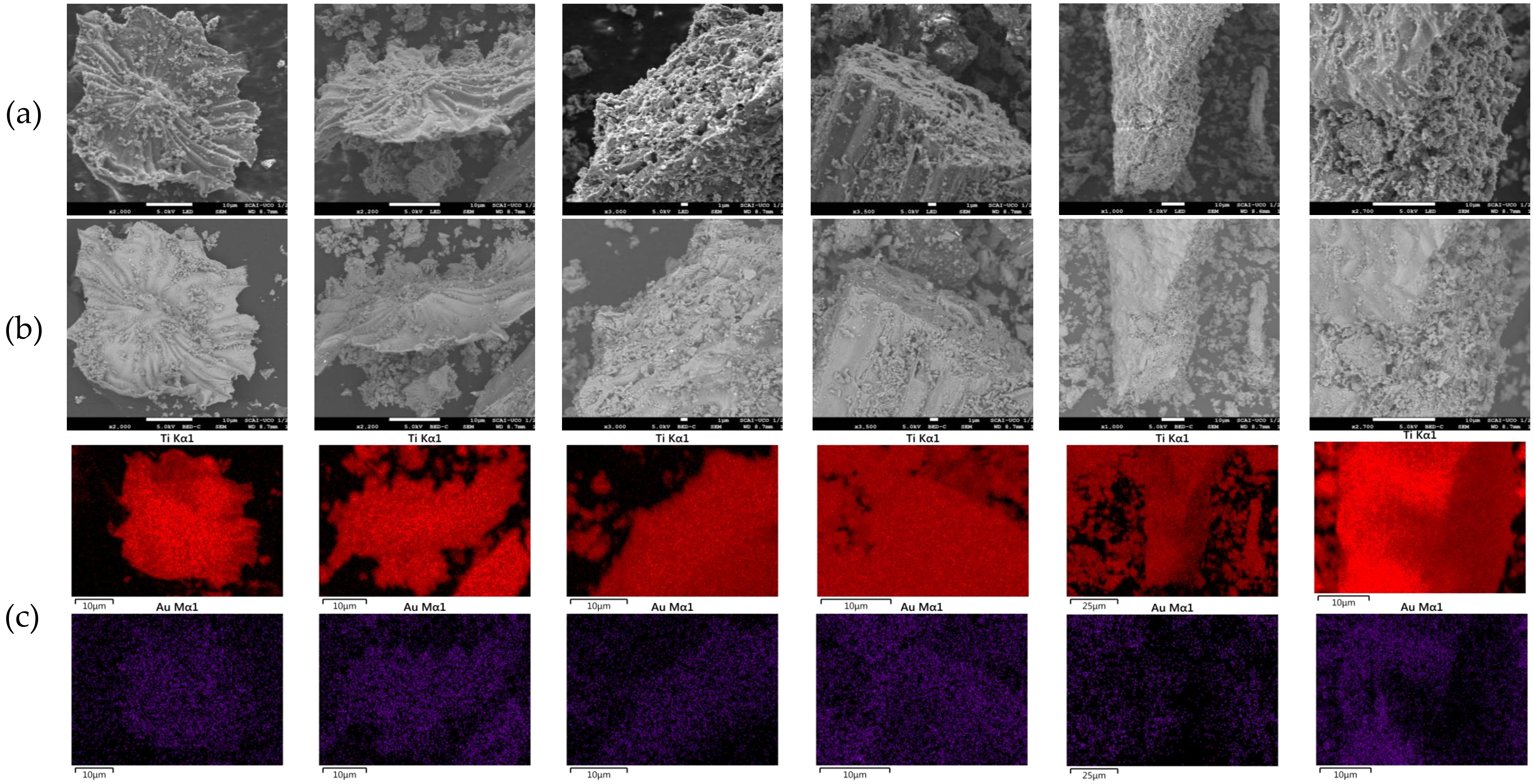

Figure S2. (a) SEM and (b) Backscattered SEM images of Au/AOL. (c) Elemental mapping (SEM-EDX) of the samples indicating the distribution of Ti and Au.

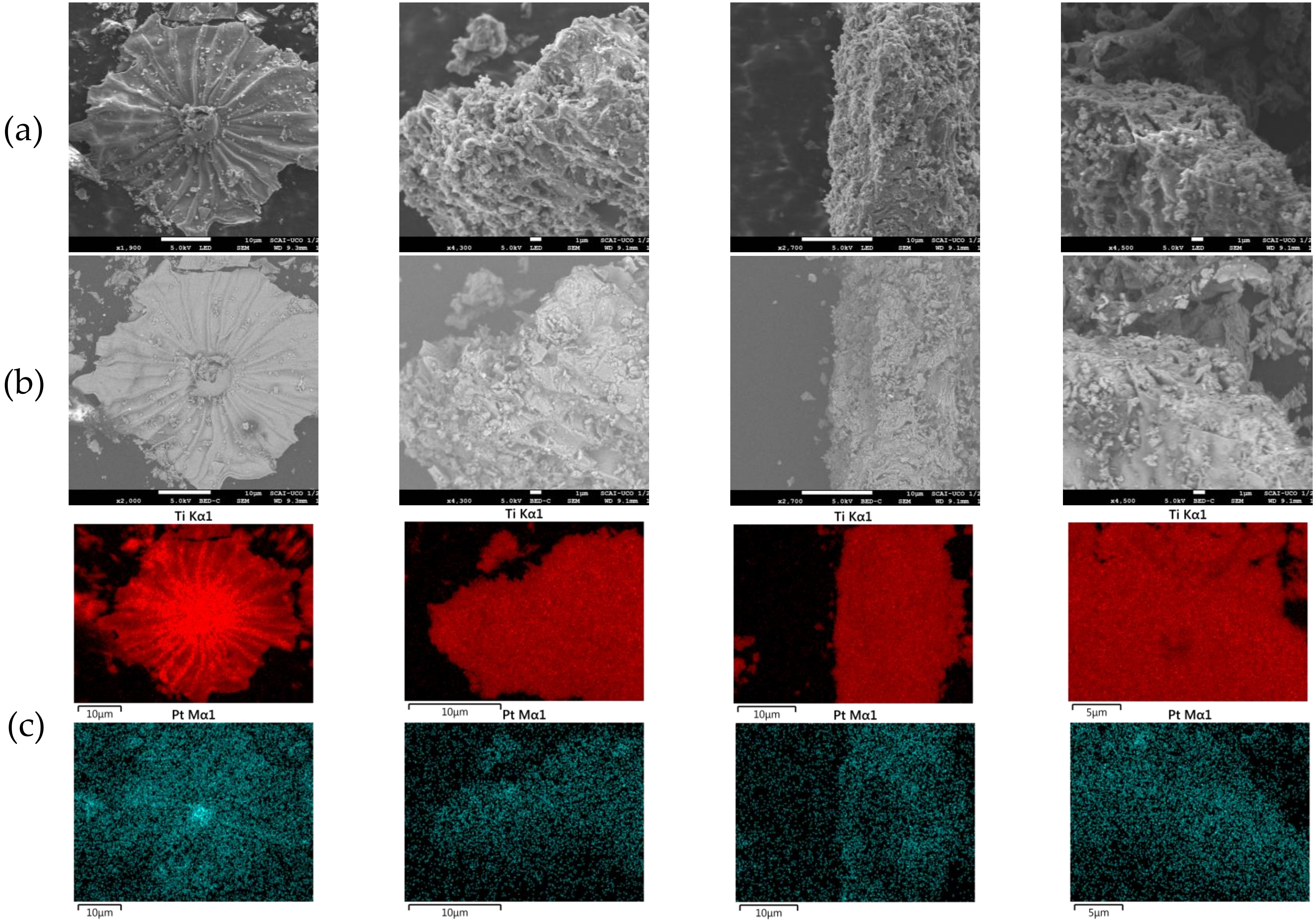

Figure S3. (a) SEM and (b) Backscattered SEM images of Pt/AOL. (c) Elemental mapping (SEM-EDX) of the samples indicating the distribution of Ti and Pt.

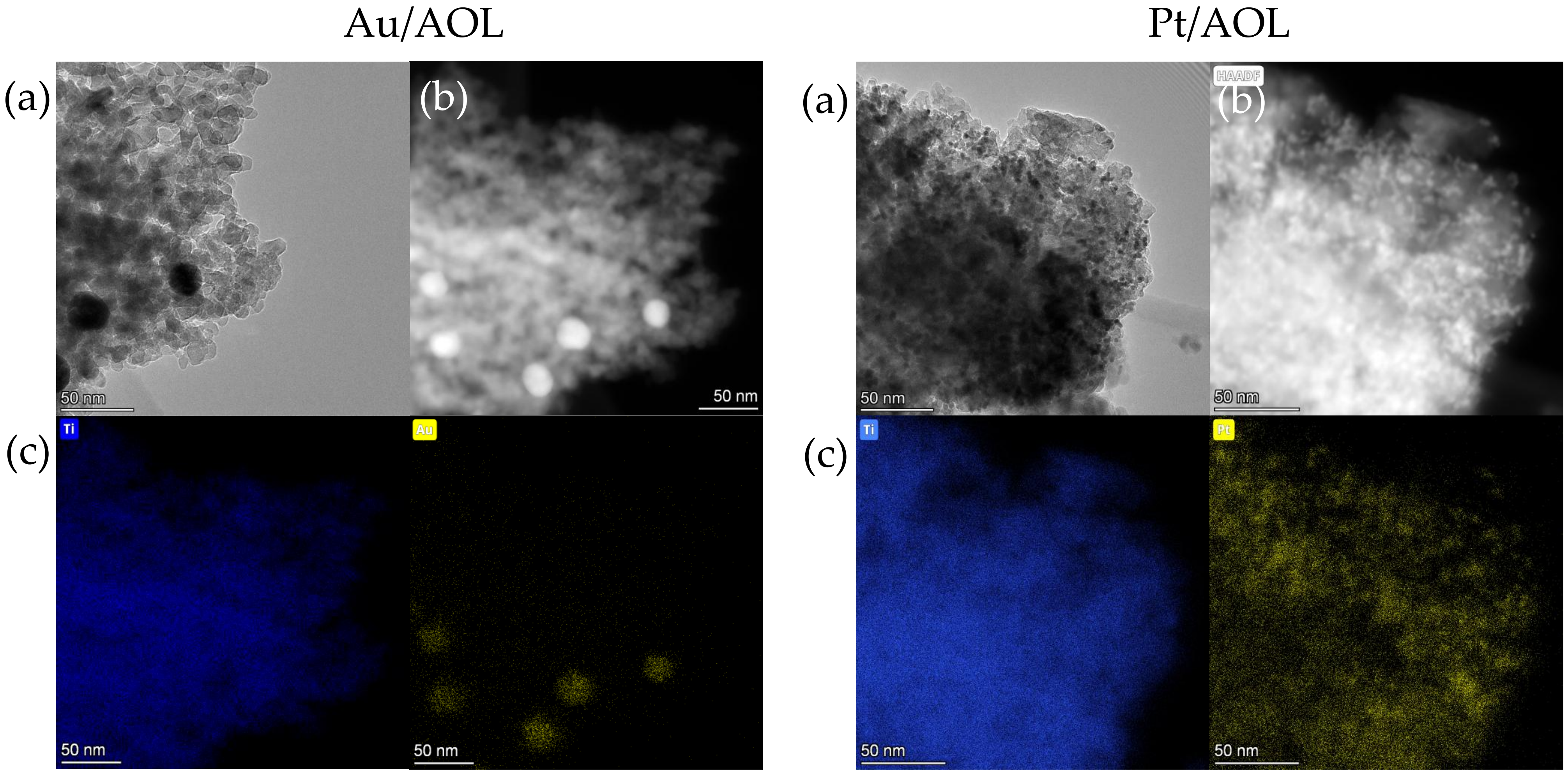

Figure S4. (a) TEM and (b) STEM images of Au/AOL and Pt/AOL. (c) Elemental mapping (STEM-EDS) of the samples indicating the distribution of Ti, Au or Pt.

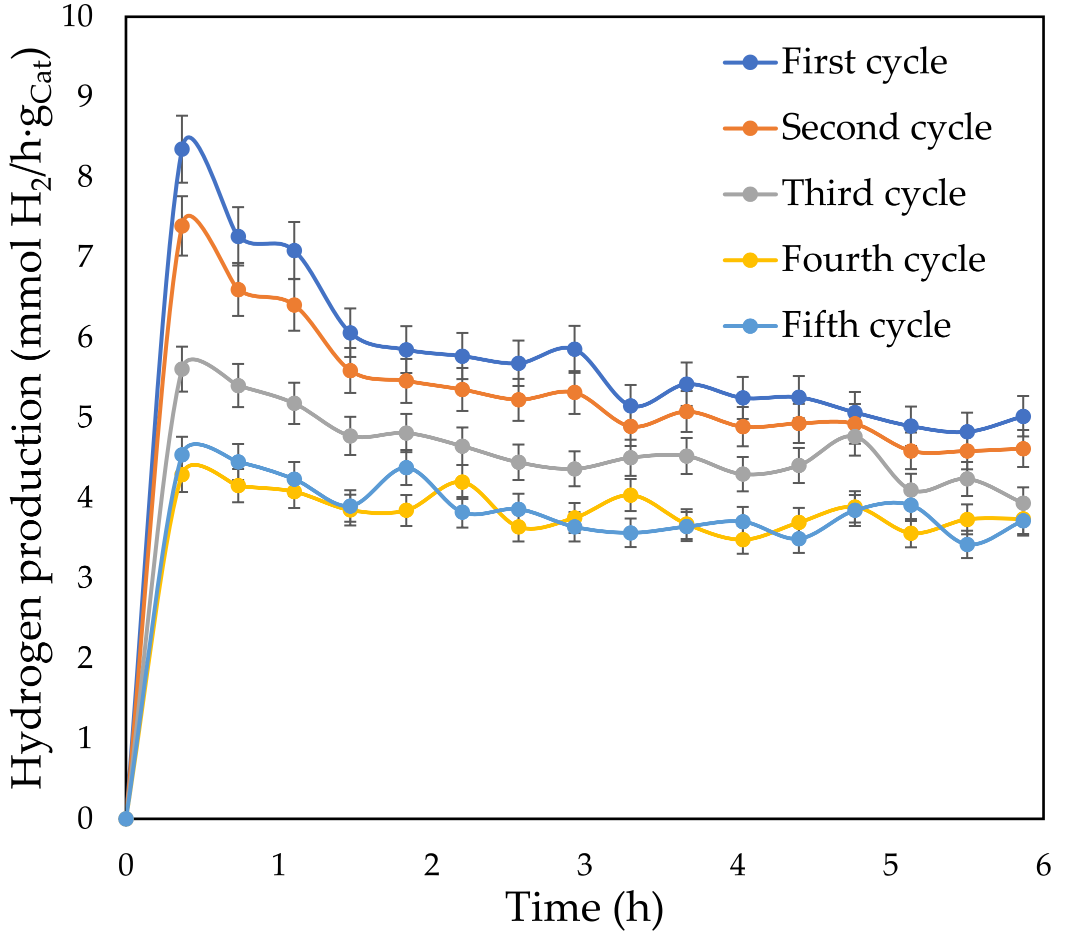

Figure S5. Hydrogen production rate over five consecutive cycles using the Pt/AOL catalyst under UV irradiation.
